# Mapping Hepatitis B Virus Research and Genomic Diversity in Latin America and the Caribbean

**DOI:** 10.3390/pathogens15101025

**Published:** 2026-09-30

**Authors:** Alexis Jose-Abrego, Sonia Roman, Irene M. Mariscal-Martinez, Karina Gonzalez-Aldaco, Arturo Panduro

**Affiliations:** 1 Departamento de Medicina Genómica en Hepatología, Hospital Civil de Guadalajara “Fray Antonio Alcalde”, Guadalajara 44280, Jalisco, Mexico; sonia.roman@academicos.udg.mx (S.R.); irene.mariscal@alumnos.udg.mx (I.M.M.-M.); karina.gonzalez@academico.udg.mx (K.G.-A.); 2 Departamento de Biología Molecular y Genómica, Centro Universitario de Ciencias de la Salud, Universidad de Guadalajara, Guadalajara 44340, Jalisco, Mexico; 3 Programa de Doctorado en Biología Molecular en Medicina, Centro Universitario de Ciencias de la Salud, Universidad de Guadalajara, Guadalajara 44340, Jalisco, Mexico; 4 Departamento de Ciencias Naturales y Exactas, Centro Universitario de los Valles, Universidad de Guadalajara, Carretera Guadalajara-Ameca Km. 45.5, Ameca 46600, Jalisco, Mexico

**Keywords:** hepatitis B virus, Latin America and the Caribbean, HBV genotypes, bibliometrics, text mining, phylogenetic analysis, molecular epidemiology, public health

## Abstract

**Background**: Hepatitis B virus (HBV) infection remains a major public health challenge in Latin America and the Caribbean (LAC). Mapping scientific production and viral genomic diversity is essential to identify consolidated research fields, as well as areas of opportunity to improve and expand research across the region. **Methods**: An integrated analysis combining bibliometrics, text-mining, and phylogenetics was performed on HBV literature indexed in PubMed, Scopus, and Web of Science (1970–2025), alongside 9528 GenBank sequences (including full-length genomes, individual genes, and partial diagnostic fragments). **Results**: A total of 4083 publications were identified, of which 72.1% (2942/4083) were from Brazil, Mexico, and Argentina. Most were original articles (75.8%; 2749/3628) involving human cohorts (95.8%; 2471/2580), where primary research fields centered on serological profiling, co-infections, vaccination, genotype characterization, and blood safety, whereas hepatocellular carcinoma, antiviral therapy, and metabolic comorbidities were the least frequently studied areas. In vitro (10.1%; 260/2580) and animal models (6.9%; 177/2580) accounted for a minor fraction of the literature. Eight circulating HBV genotypes (A–H) were detected, dominated by A (43.4%; 4138/9528), F (29.3%; 2787/9528), and D (19.8%; 1885/9528), while genotype H was largely confined to Mexico (3.2%; 306/9528). Marked geographic clustering was observed (*p* < 0.001), reflecting both autochthonous American and immigrant lineages. **Conclusions**: Epidemiological studies represent the most consolidated domain in LAC, yet research remains concentrated in a few countries with persistent experimental and clinical gaps. Fostering cross-border collaboration and investing in underexplored translational areas will be decisive in achieving regional hepatitis B elimination.

## 1. Introduction

Hepatitis B virus (HBV) remains a major global cause of liver-related morbidity and mortality, disproportionately affecting low- and middle-income regions [1]. In the Region of the Americas, HBV infection affects an estimated 2.8 million people, of whom 2.1 million live in Latin America and the Caribbean (LAC) [2,3]. The average HBV-related mortality rate in the region is 2.1 deaths per 100,000 population [2,4,5]. The highest mortality rates have been reported in Caribbean islands, including the Dominican Republic, Puerto Rico, and the Virgin Islands; in continental areas, the highest rates are observed in Bolivia and Guyana [4].

The impact of HBV in LAC is shaped by marked geographic and socioeconomic inequities [6]. Furthermore, current clinical guidelines are largely derived from studies conducted in North America, Europe, and Asia, which may limit their applicability to LAC populations [7,8,9]. At the same time, the region exhibits a complex genetic background resulting from the admixture of Indigenous American, European, and African ancestries, alongside a high diversity of HBV genotypes, including endemic variants F and H [10,11,12]. These host and viral factors interact with structural challenges—such as limited access to diagnostics, restricted treatment availability, and under-resourced healthcare systems—thereby contributing to heterogeneous patterns of disease progression and clinical outcomes. Disparities in research and diagnostic capacity may influence HBV epidemiology. Many with chronic HBV remain undiagnosed, especially in low- and middle-income countries, due to gaps in surveillance and healthcare [13,14]. These limitations may lead to underestimation of disease burden and bias data on viral diversity and outcomes, worsening regional inequities in knowledge and health efforts.

In this context, building the regional evidence base is crucial to inform effective and equitable HBV control strategies in LAC. Despite the significant burden of liver disease and the World Health Organization’s goal to eliminate viral hepatitis by 2030 [15], the HBV research landscape in LAC is fragmented and unevenly developed. It is unclear if current studies sufficiently reflect regional health priorities or address relevant clinical and experimental gaps. Furthermore, the extent to which viral genetic diversity impacts disease outcomes and informs intervention strategies has not been systematically explored in the regional context. Addressing these obstacles requires a thorough understanding of research activity, thematic priorities, and knowledge gaps across LAC. Thus, this study provides an integrated assessment of HBV research in the region through the integration of bibliometrics, text-mining and phylogenetic approaches. Specifically, we aimed to (i) analyze geographical and temporal trends in scientific production, (ii) identify thematic gaps across experimental and clinical domains, and (iii) characterize the distribution and diversity of HBV genotypes. By linking patterns of scientific production with viral genetic diversity, this study aims to inform research prioritization and to support the development of context-specific strategies for HBV control in LAC.

## 2. Materials and Methods

### 2.1. Search Strategy

**Figure 1:**
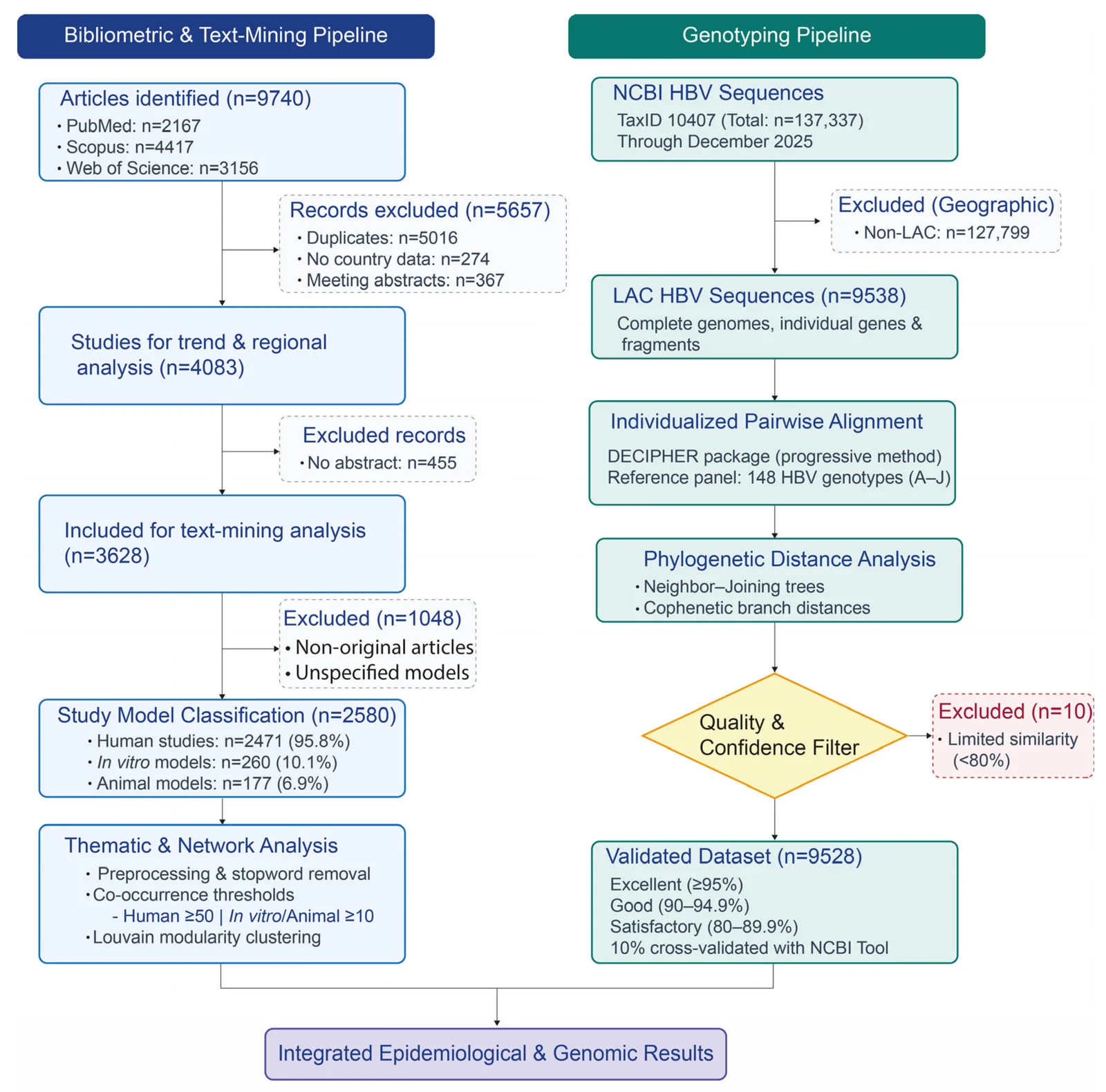
Flow diagram of article selection and HBV sequence analysis. The left panel shows the systematic screening process for published articles, including identification, application of exclusion criteria, and final inclusion for text analysis. The right panel illustrates the bioinformatic workflow applied to hepatitis B virus (HBV) sequences retrieved from GenBank.

This study was conducted using two complementary methodological approaches: (i) a systematic bibliometric review of HBV research in LAC, and (ii) phylogenetic analysis of HBV sequences retrieved from the GenBank database (Figure 1). To ensure comprehensive coverage, the literature was retrieved from three major databases: PubMed, Scopus, and Web of Science, encompassing all available publications through December 2025. The search strategy employed a combination of HBV-related terms (“hepatitis B”, “hepatitis B virus”, and “HBV”), viral antigen descriptors (hepatitis B surface antigen [HBsAg], hepatitis B core antigen [HBcAg], and hepatitis B e antigen [HBeAg]), and historical nomenclature (“Australian antigen” and “Dane particle”).

These terms were systematically paired with the names of all 47 countries and territories in the region. Geographic coverage was organized into four subregions: Mexico, Central America, South America, and the Caribbean, including both independent nations and dependent territories to ensure complete regional representation. No language restrictions were applied during database searches; publications originally written in regional languages (Spanish, Portuguese, French, and Dutch) were included, as indexed databases provide standardized English titles, abstracts, and author keywords. For all databases, article metadata—including titles, abstracts, complete author lists, institutional affiliations, journal names, publication years, and document types—were retrieved using the Rentrez package in R v1.2.3 [16] for PubMed or directly through native export tools for Scopus and Web of Science. Duplicate records were identified and removed based on article titles and, when available, PubMed identifiers (PMIDs).

### 2.2. Study Classification

After harmonizing variables and removing duplicate records, publications were classified by document type: original articles, reviews, case reports, clinical trials, meta-analyses, editorials/letters, and consensus or guideline documents. For records lacking explicit study designations, publication types were inferred using a hierarchical keyword-based classification strategy applied to title and abstract fields. The classification hierarchy was as follows: “meta-analysis” for meta-analyses; “systematic review” or “review” for reviews; “clinical trial” or “randomized controlled trial” for clinical trials; “case report” for case reports; “editorial,” “letter,” “comment,” “news,” or “newspaper” for editorials and correspondence; and “journal article,” “article,” “comparative study,” or “observational study” for original articles. Records that did not match any criteria were categorized as unclassified.

To identify experimental models, only publications classified as clinical trials, case reports, or original articles were selected for further analysis. Studies were categorized as having an identifiable research model or being unspecified based on the presence of model-specific terminology in titles and abstracts. Three model categories were defined using specific keyword sets: (i) human studies—identified by terms such as “patients,” “human,” “adults,” “children,” “infants,” “cohort,” “subjects,” “volunteers,” “clinical,” “serum,” “plasma,” and “blood samples”; (ii) in vitro studies—identified by “Madin-Darby canine kidney cells,” “*Pichia pastoris*,” “cell line,” “in vitro,” “apoptosis,” “hepatocytes,” “co-transfection,” “yeast,” “HepG2,” “cysteine,” “expression,” “transgenic tobacco plants,” and “mammalian cells”; and (iii) animal studies—identified by “chimpanzees,” “mice,” “rats,” “primates,” “animal model,” “avian,” and “bat.”

### 2.3. Text Mining Analysis

A text mining analysis was conducted to identify thematic patterns across human, animal, and in vitro studies. For each category, article titles and abstracts were concatenated to form a unified text corpus; publications lacking abstracts were excluded. Text preprocessing was performed in R using the tidytext (v0.3.1), stringr (v1.5.0), and dplyr (v1.1.2) packages [17,18,19]. The combined text corpus was tokenized into individual words, followed by systematic removal of non-informative terms across four predefined categories: (i) statistical terminology (median, mean, *p*-value, confidence interval, standard deviation, significance, regression, odds ratio); (ii) generic domain-related vocabulary (polymerase, chain, copies, amplification, international units, serum, samples); (iii) common function words and connectors (and, the, in, with, of, to, by, on, for, from, between); and (iv) standard English stopwords (is, was, were, this, that, it, are, has, have, had). In addition, all numerical characters and punctuation marks were removed.

After text cleaning, word frequency distributions were calculated separately for each study model. To capture semantic relationships, bigrams (two-word sequences) were extracted, and bigram frequency matrices were constructed for each category. Co-occurrence networks were generated using the igraph (v1.3.5) and ggraph (v2.1.0) packages [20,21], with bigrams filtered using model-specific frequency thresholds (≥50 occurrences for human studies and ≥10 occurrences for animal and in vitro studies) to balance interpretability and coverage. Networks were represented as undirected graphs, in which nodes corresponded to individual terms and edges represented bigram co-occurrence. Node size was scaled according to term frequency, defined as the cumulative number of bigram occurrences containing each term, while edge width and transparency were mapped to bigram frequency using a log_10_ transformation to account for right-skewed distributions. Semantic community detection was performed using the Louvain modularity optimization algorithm, with identified clusters subsequently ranked and renumbered in descending order of cumulative word frequency. Network topologies were computed using the Fruchterman–Reingold force-directed layout algorithm over 10,000 iterations. To ensure complete algorithmic determinism and spatial reproducibility across runs, fixed pseudo-random seeds were established prior to layout generation: set.seed(123) for human studies, set.seed(456) for in vitro models, and set.seed(789) for animal models. Both algorithms and spatial projections were executed via the igraph (v1.3.5) and ggraph (v2.1.0) packages in R [20,21].

### 2.4. HBV Genotyping and Subgenotyping

To perform the phylogenetic analysis of HBV in LAC, sequences reported through December 2025 were obtained from the NCBI Virus resource (Taxonomy ID: 10407; https://www.ncbi.nlm.nih.gov/labs/virus/vssi/#/ (accessed on 15 December 2025) from an initial pool of 137,337 global records. Based on geographic metadata, 9538 sequences from LAC were filtered and downloaded in FASTA format (including full-length genomes, individual genes, and partial diagnostic fragments).

Due to HBV sequence heterogeneity, a comprehensive multiple-sequence alignment of all sequences against reference genomes was not feasible. We therefore implemented an individualized genotyping approach in which each HBV sequence was aligned independently against a curated reference panel of 148 full-length HBV genomes (~3266 bp) representing genotypes A through J with multiple representatives per subgenotype. Multiple sequence alignments were performed using a progressive alignment algorithm in the DECIPHER R package v2.24.0 [22]. Pairwise genetic distances were calculated using the raw proportional distance method. Phylogenetic trees were constructed using the neighbor-joining method, and cophenetic distances (branch-length distances in the tree) were computed to identify the closest reference sequences; both analyses used the ape R package v5.8 [23]. Genotype assignments were categorized by similarity into four confidence levels: Excellent (≥95%), Good (90.0–94.9%), Satisfactory (80.0–89.9%), and Limited (<80.0%). Only sequences with Satisfactory or higher confidence were included in downstream analyses. Methodological accuracy was validated by independently genotyping a randomly selected subset of 10% of the sequences (*n* = 954) using the web-based NCBI HBV Genotyping Tool (https://www.ncbi.nlm.nih.gov/projects/genotyping/formpage.cgi (accessed on 15 December 2025), which yielded a 100% concordance rate in genotype classification. The accession numbers of the reference sequences, together with the genotype and subgenotype assignments for all analyzed samples (*n* = 9528), are provided in Supplementary Table S1.

### 2.5. Statistical Analysis

Descriptive statistics were used to summarize publication counts by country, year, journal, publication type, HBV genotype/subgenotype, and study model (human, animal, or in vitro). Categorical variables were reported as frequencies and proportions. For the text mining component, the most frequent terms and bigrams within each study model were identified using a frequentist approach [21]. All statistical and text mining analyses were performed using R software (v4.5.1, Great Square Root, R Foundation for Statistical Computing, Vienna, Austria). Statistical significance was defined as a two-tailed *p* < 0.05.

## 3. Results

### 3.1. Geographic Distribution and Temporal Trend of Hepatitis B Research

**Figure 2:**
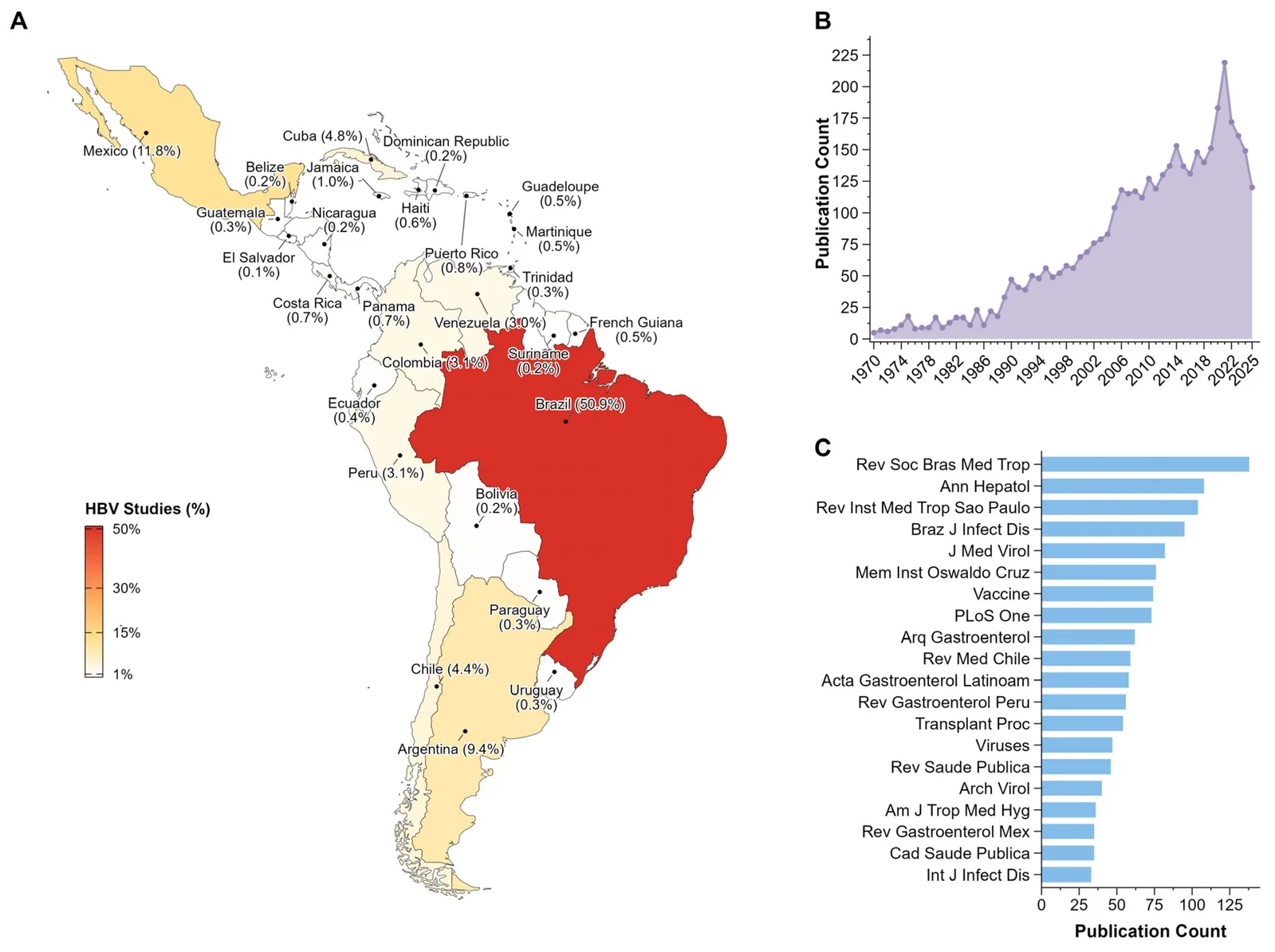
Spatial and temporal distribution of hepatitis B research in Latin America and the Caribbean. (**A**) Proportion of hepatitis B studies by country; percentages are calculated relative to total regional publications (*n* = 4083), and countries/territories with 0.0% are not labeled for visual clarity. (**B**) Temporal evolution of publications over time. (**C**) Top 20 journals in which the included hepatitis B studies were published.

The geographic distribution of HBV research output across LAC is summarized in Figure 2A. Country-of-origin data were available for 4083 publications published between 1970 and 2025. Brazil accounted for half of the total output (50.9%; 2078/4083), followed by Mexico (11.8%; 482/4083) and Argentina (9.4%; 382/4083). Together, these three countries contributed 72.1% of the regional literature. Intermediate levels of productivity were observed in Cuba (4.8%), Chile (4.4%), Colombia (3.1%), Peru (3.1%), and Venezuela (3.0%). In contrast, Central American and Caribbean territories accounted for lower proportions of indexed records (<0.1% each).

The temporal evolution of HBV research in LAC is shown in Figure 2B. Annual publication counts remained low during the 1970s (<20 articles per year) and increased gradually throughout the 1980s and 1990s, surpassing 50 publications in 1993. After 2000, research productivity increased markedly, exceeding 100 publications per year in 2005 and 150 by 2014. Peak output was reached in 2021, with 219 publications. A subsequent decline was observed, with 161 publications in 2023, 149 in 2024, and 120 in 2025.

The distribution of HBV publications across journals is shown in Figure 2C. The top 20 journals accounted for 32.6% (1331/4083) of all identified articles, spanning regional and international publication venues. The most represented journals were *Revista da Sociedade Brasileira de Medicina Tropical* (3.4%; 138/4083), *Annals of Hepatology* (2.6%; 108/4083), and *Revista do Instituto de Medicina Tropical de São Paulo* (2.5%; 104/4083). International journals were also well represented, including the *Journal of Medical Virology* (2.0%; 82/4083), *Vaccine* (1.8%; 74/4083), and *PLoS One* (1.8%; 73/4083).

### 3.2. Types of Publications in Hepatitis B Research

**Figure 3:**
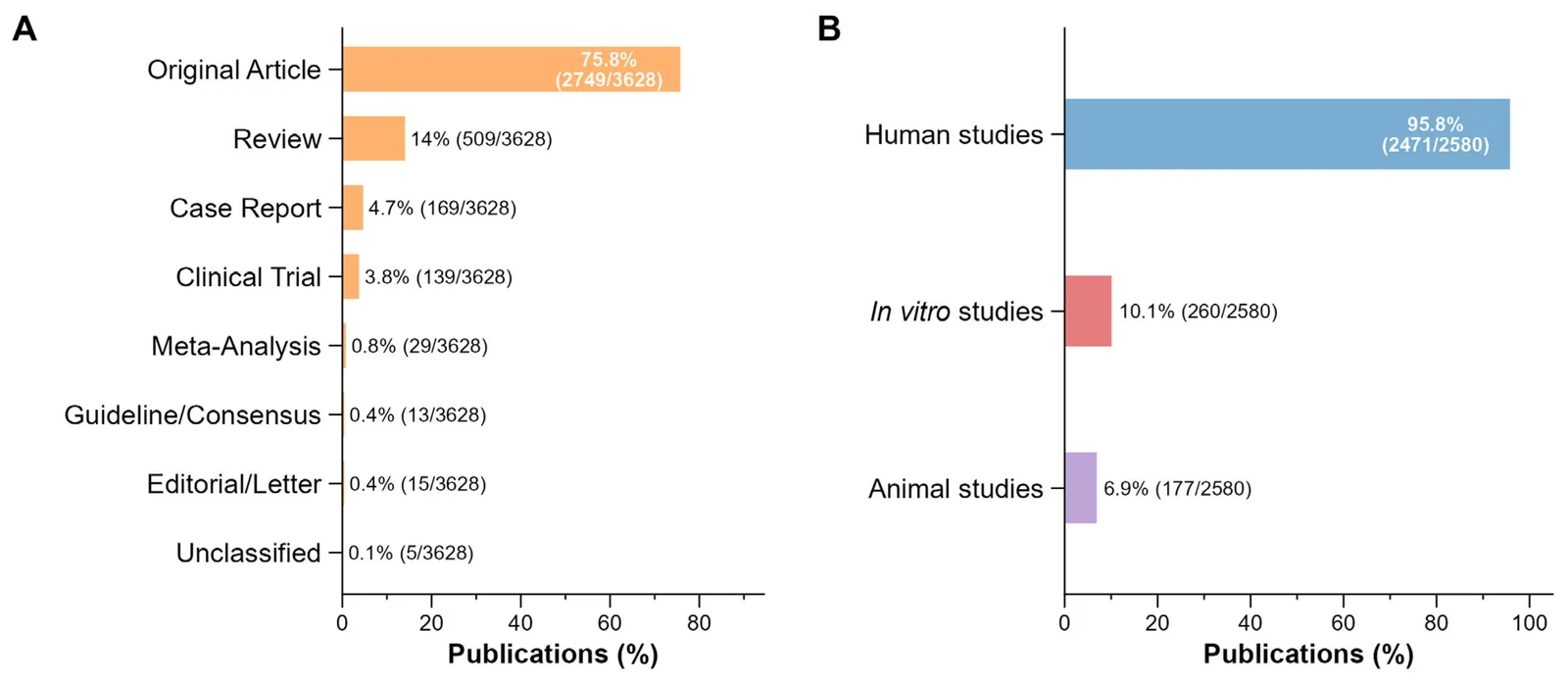
Distribution of hepatitis B publications by article type and study model. (**A**) Proportion of publications by publication type. (**B**) Distribution of studies by experimental model. In panel (**B**), percentages exceed 100% because certain publications employed hybrid or multiple experimental models.

Out of the 4083 publications that were found, 88.9% (3628/4083) had abstracts and could be classified as content (Figure 3A). Original articles accounted for the majority of publications (75.8%; 2749/3628), followed by reviews (14.0%; 509/3628), case reports (4.7%; 169/3628), and clinical trials (3.8%; 139/3628). Other publication types, including meta-analyses, editorials or letters, and guidelines, collectively represented 1.6% (57/3628), whereas only 0.1% (5/3628) remained unclassified.

Original articles, case reports, and clinical trials (*n* = 3057) were incorporated into the study-type analysis to further describe the research design. Of these, 84.4% (2580/3057) offered enough methodological information to be classified (Figure 3B). Human studies predominated, accounting for 95.8% (2471/2580) of publications. In vitro and animal studies represented 10.1% (260/2580) and 6.9% (177/2580), respectively. Percentages exceed 100% because some studies used multiple experimental models.

### 3.3. Principal Thematic Structure of Hepatitis B Research in Latin America and the Caribbean

**Figure 4:**
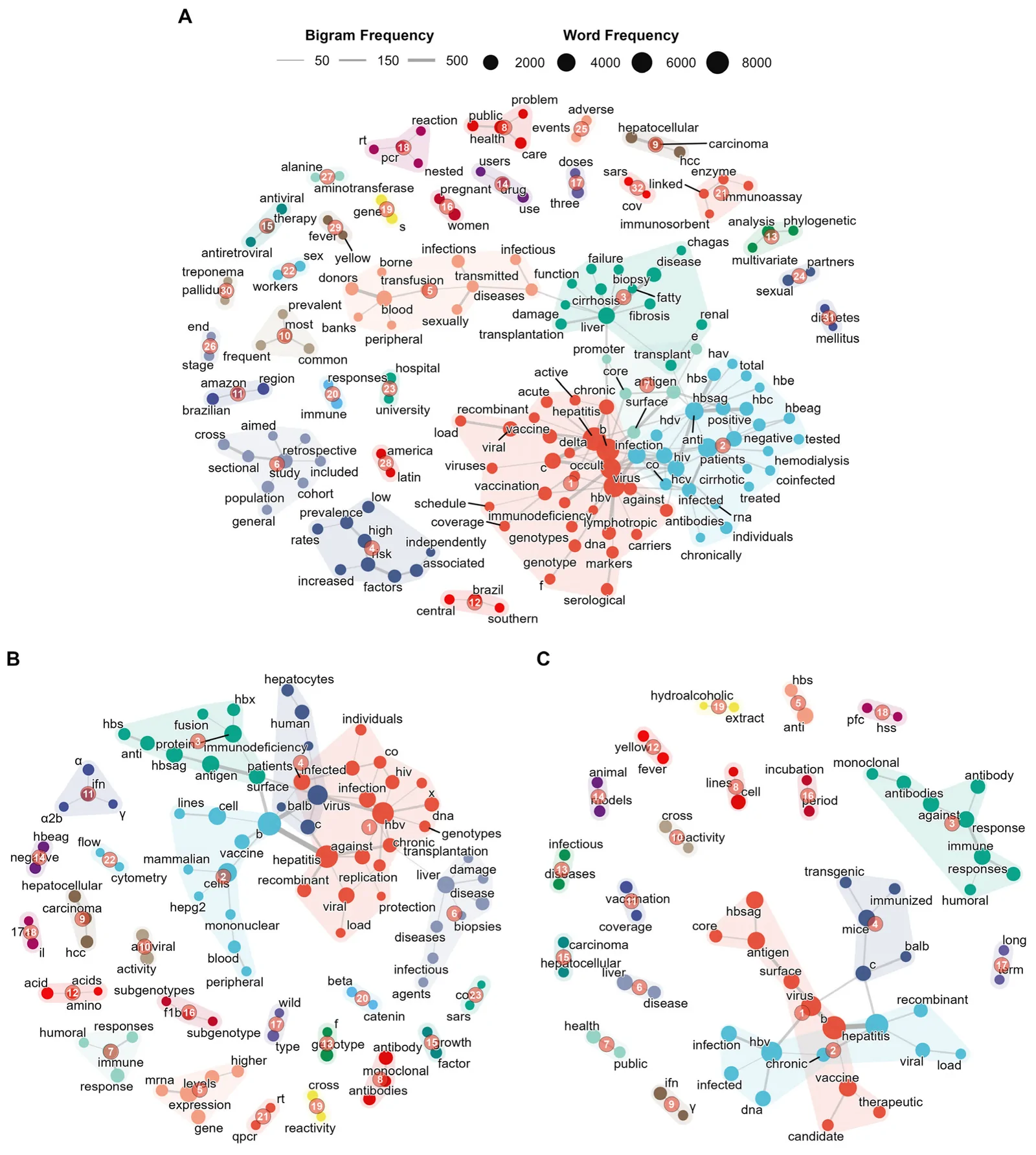
Bigram network analysis of HBV publications stratified by experimental model. (**A**) Human studies (co-occurrence threshold ≥ 50), (**B**) in vitro studies (threshold ≥ 10), and (**C**) animal studies (threshold ≥ 10). Node size reflects individual keyword frequency, while edge width and transparency indicate bigram co-occurrence strength (log_10_—scaled). Shaded convex polygons delineate semantic clusters identified by the Louvain modularity algorithm, numbered sequentially (1 to *k*) in descending order of total cluster weight. Numbered red badges mark cluster centroids.

Text-mining analysis revealed clear differences in the thematic structure of HBV research across experimental models (Figure 4). Human-based studies (Figure 4A) generated 32 clusters, with the six largest accounting for most of the conceptual landscape. Cluster 1 was the most prevalent (36.54%; 49,283/134,882), mainly focusing on core virology, DNA characterization, and vaccination strategies, featuring terms such as *hepatitis*, *HBV*, *vaccine*, *DNA*, *vaccination*, *genotypes/genotype f*, and *occult infection*. Cluster 2 (13.64%; 18,393/134,882) centered on the clinical reality of co-infection, grouping terms such as *patients*, *HCV*, *HIV-infected*, *coinfected*, and *hemodialysis*. Cluster 3 (12.75%; 17,202/134,882) was dedicated to serological profiling and diagnostics (*anti*, *HBsAg*, *antigen*, *HBc*, *surface*, and *HDV*). Cluster 4 (5.77%; 7776/134,882) encompassed clinical outcomes and liver pathology (*cirrhosis*, *transplantation*, *fibrosis*, *biopsy*, and the regional comorbidity *Chagas*). Cluster 5 (5.76%; 7765/134,882) reflected epidemiological determinants, with terms such as *risk*, *prevalence*, *factors*, *increased*, and *rates*. Cluster 6 (5.19%; 7003/134,882) specifically addressed blood safety and transmission routes, as evidenced by terms like *blood*, *donors*, *banks*, *transfusion*, *sexually transmitted*, and *borne infections*.

In vitro studies (Figure 4B) comprised 23 clusters. Cluster 1 (29.70%; 3398/11,440) focused on viral replication and its interaction with other pathogens (*HBV*, *infection*, *replication*, *load*, *HIV*, and *recombinant*). Cluster 2 (17.01%; 1946/11,440) comprised terms related to laboratory cell systems, including *cells*, *cell lines*, *mammalian*, *HepG2*, and *peripheral blood mononuclear* cells. Cluster 3 (12.96%; 1483/11,440) was related to viral protein characterization, including *HBsAg*, *surface*, *antigen*, *fusion*, and the oncogenic *HBx* protein. Cluster 4 (8.26%; 945/11,440) highlighted the use of human-derived cellular models, involving *human hepatocytes* and *immunodeficiency* contexts. Cluster 5 (7.03%; 804/11,440) was linked to molecular biology and transcriptional activity (*expression*, *gene*, *mRNA*, and *levels*). Cluster 6 (5.68%; 650/11,440) represented research into tissue-level damage and disease mechanics, with terms including *liver disease*, *damage*, *agents*, and *biopsies*.

Animal-model studies (Figure 4C) were consolidated into 19 clusters, with the top four defining the pre-clinical research pipeline. Cluster 1 (29.23%; 1751/5990) was primarily associated with the development of prophylactic and therapeutic tools (*vaccine*, *candidate*, *therapeutic*, *antigen*, and *HBsAg*). Cluster 2 (25.57%; 1532/5990) was focused on viral dynamics and molecular infection markers (*hepatitis*, *HBV*, *infection*, *DNA*, *viral load*, and *recombinant*). Cluster 3 (14.44%; 865/5990) was related to the characterization of the immune response, particularly *humoral* and *monoclonal antibody* production. Cluster 4 (6.98%; 418/5990) reflected the standardization of laboratory models, specifically the use of *transgenic* and *BALB/c mice*.

### 3.4. Complex Regional Patterns of HBV Genotype and Subgenotype Diversity in LAC

**Figure 5:**
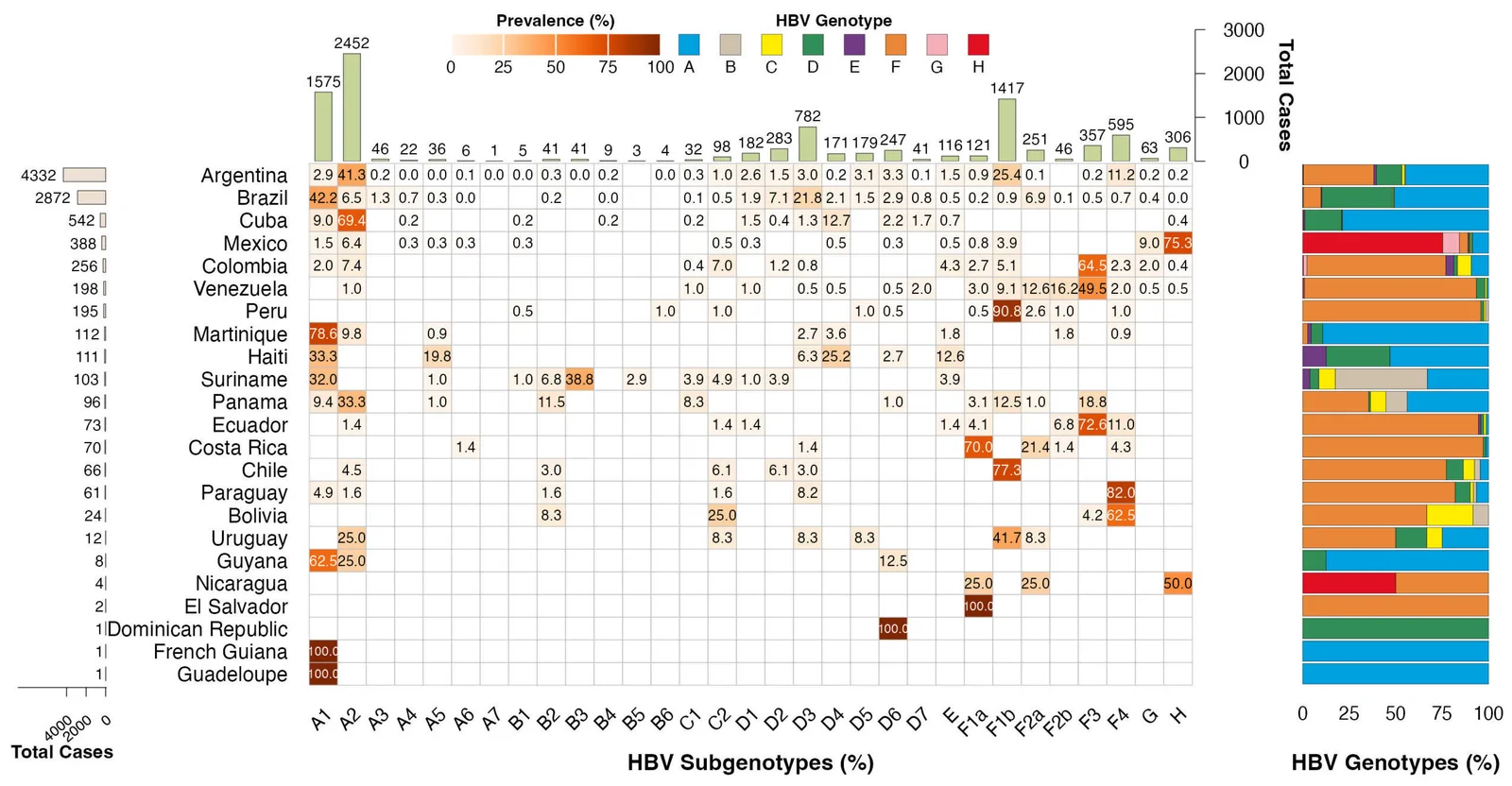
Geographic distribution and genetic diversity of hepatitis B virus in Latin America and the Caribbean. The left panel indicates the number of HBV sequences deposited in GenBank by country; the central panel shows country-level proportions of HBV subgenotypes; the right panel summarizes HBV genotype composition; and the top panel displays overall subgenotype abundance.

A total of 9528 HBV sequences from LAC were retrieved from GenBank (Figure 5). The sequences were released between January 1998 and December 2025. Sequence availability was highly heterogeneous across countries, with Argentina (45.5%; 4332/9528) and Brazil (30.2%; 2872/9528) together accounting for 75.6% of all sequences (7204/9528), compared with 24.4% (2324/9528) from the remaining countries combined. Eight HBV genotypes (A–H) were detected. Genotype A was the most prevalent (43.4%; 4138/9528), followed by F (29.3%; 2787/9528), D (19.8%; 1885/9528), and H (3.2%; 306/9528); genotypes C (1.4%; 130/9528), E (1.2%; 116/9528), B (1.1%; 103/9528), and G (0.7%; 63/9528) were detected at lower frequencies. Subgenotype distribution varied significantly across countries (*p* < 0.001).

Within genotype A, subgenotypes A2 (59.2%; 2452/4138) and A1 (38.1%; 1575/4138) predominated. Expressed as a proportion of each country’s total sequences, A2 was most frequent in Cuba (69.4%; 376/542), Argentina (41.3%; 1791/4332), and Panama (33.3%; 32/96), whereas A1 predominated in Martinique (78.6%; 88/112), Brazil (42.2%; 1211/2872), and Haiti (33.3%; 37/111). Within genotype F, half of the sequences (50.8%; 1417/2787) belonged to subgenotype F1b, followed by F4 (21.3%; 595/2787) and F3 (12.8%; 357/2787). F1b was especially prevalent in Peru (90.8%; 177/195) and Chile (77.3%; 51/66). Subgenotype F4 was most often detected in Paraguay (82.0%; 50/61) and Bolivia (62.5%; 15/24), while F3 was dominant in Ecuador (72.6%; 53/73), Colombia (64.5%; 165/256), and Venezuela (49.5%; 98/198). In genotype D, the most common subgenotypes were D3 (41.5%; 782/1885), D2 (15.0%; 283/1885), and D6 (13.1%; 247/1885). D4 was also frequently found in Haiti (25.2%; 28/111) and Cuba (12.7%; 69/542). D3 was predominantly detected in Brazil (21.8%; 625/2872), Paraguay (8.2%; 5/61), and Haiti (6.3%; 7/111). D2 was most frequently identified in Brazil (7.1%; 204/2872) and Chile (6.1%; 4/66), while D6 was primarily observed in Argentina (3.3%; 144/4332) and Brazil (2.9%; 82/2872). Although the Dominican Republic and Guyana showed proportional detection of D6, the absolute number of sequences from these countries was limited and should be interpreted with caution. Genotype H was largely restricted to Mexico (75.3%; 292/388), with limited detection elsewhere. Among minor genotypes, genotype C included subgenotypes C2 (75.4%; 98/130) and C1 (24.6%; 32/130). C2 was more common in Bolivia (25.0%; 6/24), Colombia (7.0%; 18/256), and Argentina (1.0%; 45/4332), while C1 was predominantly found in Panama (8.3%; 8/96) and Suriname (3.9%; 4/103). Genotype E was detected in Haiti (12.6%; 14/111), Colombia (4.3%; 11/256), Suriname (3.9%; 4/103), and Argentina (1.5%; 63/4332). Subgenotypes B3 and B2 comprised equal proportions of genotype B (39.8% each; 41/103), with B3 being most prevalent in Suriname (38.8%; 40/103) and B2 identified across Panama (11.5%; 11/96), Bolivia (8.3%; 2/24), and Suriname (6.8%; 7/103). Finally, genotype G was most frequently detected in Mexico (9.0%; 35/388), Colombia (2.0%; 5/256), Brazil (0.4%; 12/2872), and Argentina (0.2%; 10/4332).

## 4. Discussion

This study provides an integrated assessment of HBV research in LAC and identifies three key findings. First, HBV research is concentrated in a limited number of countries, which may reflect underlying inequalities in research capacity across the region. Second, research was predominantly oriented toward serological and observational epidemiology, whereas basic experimental models and specific clinical domains—such as hepatocellular carcinoma, antiviral therapy, and metabolic comorbidities—had a lower relative representation. Third, HBV in LAC exhibits high genetic diversity, with non-A genotypes predominating and subgenotypes showing clear geographic structuring. Together, these findings suggest a mismatch between patterns of research activity, regional health needs, and the underlying viral landscape, which may pose challenges to current HBV control and elimination efforts.

The historical evolution of HBV research in LAC provides important context for understanding current structural inequalities. Early investigations in the 1970s were conducted in Mexico, Chile, and Brazil when HBV infection was still referred to as the “Australia antigen” [24,25,26]. In Mexico, Salazar-Mallén et al. documented HBs antigen prevalence in patients with lepromatous leprosy and those with Down syndrome [25,26]. Sepúlveda et al. later found a prevalence of 0.66% among blood donors [27]. In Chile, Velasco and Katz described the HBs antigen in different clinical situations and related it to chronic liver disease [28,29]. In Brazil, Salzano and Blumberg found the antigen in healthy individuals and in patients with leprosy or leukemia [30].

Despite these early contributions, research activity in the region remained limited until the mid-1980s, likely reflecting technological and economic constraints, especially the high cost and limited availability of immunodiffusion assays, which were among the main diagnostic tools available at the time [31]. Between 1987 and 2021, publication output grew steadily alongside advances in diagnostic and molecular technologies, notably ELISA for HBV surface antigen detection and PCR for viral DNA analysis, which facilitated research across diverse settings [32,33,34]. These advances enabled the identification of HBV genotypes A–D in 1988, with endemic genotypes F and H discovered in the 1990s and 2002, respectively [35,36,37,38]. Increased recognition of HBV’s role in hepatocellular carcinoma contributed to greater public health attention and research activity [39]. The 2021–2024 decline in publications may reflect shifts in research funding toward COVID-19, perceptions of control through vaccination in high-income countries, and ongoing challenges in low- and middle-income countries, where transmission, diagnosis, and treatment issues persist [40,41,42,43]. Together, these dynamics may help explain the current uneven patterns of research activity and persistent knowledge gaps across LAC [44].

Our study identified a strong concentration of HBV research in LAC, with more than 70% of publications originating from Brazil, Mexico, and Argentina. This pattern suggests that large parts of the region may be underrepresented in epidemiological and clinical literature. Brazil’s prominent position is likely associated with sustained investment in science and technology, with research expenditure reaching 1.19% of GDP in 2023—one of the highest rates in LAC—compared with 0.60% in Argentina and 0.25% in Mexico [45,46]. This investment is further supported by strong institutional frameworks, including organizations such as the São Paulo Research Foundation (FAPESP), the National Council for Scientific and Technological Development (CNPq), and the Coordination for the Improvement of Higher Education Personnel (CAPES), which fund long-term research and graduate training programs [47,48,49,50]. These structural advantages are also reflected in publication patterns, including the prominence of national journals such as *Revista da Sociedade Brasileira de Medicina Tropical* and *Revista do Instituto de Medicina Tropical de São Paulo*. At the same time, international journals—including *Journal of Medical Virology*, *Vaccine*, and *PLoS One*—featured prominently, suggesting a dual regional–international dissemination strategy [51]. Together, sustained investment, strong institutional support, and access to indexed publication platforms may help explain Brazil’s leading contribution to HBV research in LAC. Addressing these imbalances, however, will require not only increased funding but also stronger cross-border collaboration. In this context, partnerships with the region’s most productive research groups (Supplementary Figure S1) could help expand research activity and strengthen collaborative networks. Promoting research exchanges and training programs may facilitate the transfer of technical expertise and the standardization of protocols across the region.

Our text-mining analysis identified distinct patterns across HBV research fields in LAC. Observational and epidemiological studies in human populations represented the most consolidated fields, particularly research on serological profiling, co-infections, hepatitis B vaccination, genotype and subgenotype characterization, liver pathology, and transfusion safety. The prominence of these fields may reflect several interrelated factors, including the urgent public health need to prevent viral transmission and ensure blood safety in endemic and vulnerable populations [52]; growing interest in evaluating vaccination coverage as hepatitis B immunization programs expand [8]; the feasibility of cross-sectional designs using routinely collected hospital and laboratory data [53]; and differences in infrastructure and technical capacity that may favor population-level surveillance over basic experimental research [46,54]. These observational studies provide essential evidence for characterizing the burden and distribution of HBV infection, identifying populations at increased risk, monitoring vaccination programs, describing circulating viral genotypes and clinical manifestations, and informing strategies for disease prevention and control [52].

Conversely, the least-represented HBV research fields were hepatocellular carcinoma (HCC), antiviral therapy, and comorbidities such as diabetes, obesity, and nutritional management. Expanding research in these areas is particularly relevant given the rising regional burden of metabolic dysfunction-associated steatotic liver disease (MASLD) in LAC, estimated at 44.4% [55,56]. This burden is driven by the displacement of traditional native diets by ultra-processed foods, alongside sedentary lifestyles and escalating rates of type 2 diabetes, together fostering a pronounced obesogenic environment [57]. Host genetic variation could further compound this metabolic scenario. Notably, the region has the highest prevalence worldwide of the *patatin-like phospholipase domain-containing protein 3* (*PNPLA3*) p.I148M risk allele (rs738409), which is associated with both MASLD and HCC in patients with cirrhosis [58,59]. In regional cohorts, such as in Mexico, the *PNPLA3* GG genotype confers a 2.24-fold increased risk of elevated alanine aminotransferase (ALT) levels, indicating heightened susceptibility to hepatic necroinflammation [60]. In addition to host factors, viral genetic diversity may also influence liver disease progression in the region; notably, HBV subgenotype F1b is prevalent in several South American populations and has been detected in patients with HCC, particularly in Peru, compounding the risk of HBV-related cirrhosis and liver cancer [61]. Together, these findings highlight the need for an integrated research approach to determine how host factors (genetic and metabolic), the obesogenic environment, and HBV genotype diversity influence the progression of liver disease across LAC countries.

We also found that basic experimental research was the least represented domain in LAC, with in vitro studies accounting for 10.1% and animal models for 6.9%. This pattern may have important implications for regional research development, as these experimental platforms constitute a critical preclinical pipeline for developing regionally relevant antiviral therapies and for discovering and validating nutrients and nutritional interventions with potential anti-HBV activity [62,63,64,65,66]. Without sufficient local preclinical research, HBV care in LAC may become increasingly reliant on discoveries and therapeutic strategies developed in North America, Europe, and Asia [7,8,9], potentially limiting the region’s capacity to develop clinical and nutritional strategies tailored to its populations. Strengthening basic research capacity and technical expertise in LAC is therefore essential to expand the regional evidence base and complement existing epidemiologic and clinical research, ultimately supporting comprehensive HBV prevention, treatment, and elimination efforts [2,3].

At the same time, studies on viral genetic diversity provide other perspectives on the regional HBV scenario. Analysis of HBV sequences deposited in GenBank (1998–2025) revealed substantial genotype/subgenotype diversity across LAC, with marked geographic variation in their distribution. Lineages associated with indigenous populations—particularly genotypes F and H—were regionally predominant and showed clear geographic clustering consistent with early human migration into the Americas and subsequent long-term isolation and host–virus co-evolution [11,12,67]. Genotype F showed the most pronounced geographic structuring: subgenotype F1b predominated in Peru and Chile, F3 in Ecuador and Colombia, F4 in Paraguay, Bolivia, and Argentina, and F1a was highly represented in Costa Rica. Genotype H, by contrast, was observed almost exclusively in Mexico. Together, these patterns are consistent with prior evidence that genotypes F and H are largely restricted to the Americas and closely associated with indigenous populations [11,12,68], with their subgenotypic divergence likely reflecting ancient viral lineages introduced by early human populations and shaped by subsequent geographic isolation over millennia [69,70].

HBV genotypes associated with European lineages, particularly in the Southern Cone, showed substantial prevalence. Subgenotype A2 was common in Argentina and Cuba, while genotype D (D2, D3, D6) was widespread in Argentina, Brazil, and Uruguay. Globally, A2 dominates Northern and Western Europe, and D in Southern Europe and the Mediterranean [71]. These patterns are consistent with HBV lineages introduced during Spanish and Portuguese colonization and likely amplified by European immigration in the 19th and 20th centuries [72]. Meanwhile, HBV genotypes associated with African lineages showed regional clustering, notably in Brazil and the Caribbean, where subgenotype A1 and genotype E predominated. Haiti had a distinct profile with a high co-prevalence of A1, E, and D4, likely linked to the transatlantic slave trade and forced displacement from West and Central Africa [73]. Recent evidence suggests ongoing introduction of genotype E into the Americas, including Mexico and Colombia [74,75], with contemporary migration likely influencing its distribution.

Genotypes associated with Asian lineages, particularly B and C, are observed at lower frequencies but show localized enrichment. B3 is concentrated in Suriname, and C2 in Bolivia and Panama. These patterns are consistent with HBV lineages introduced through labor migrations from East and Southeast Asia since the late 1800s. Japanese labor migration to Peru (1899) and Brazil (1908) contributed to the introduction and regional dissemination of these lineages [76]. Phylogenetic studies suggest that genotypes B and C identified in Bolivia cluster with Asian lineages and are likely linked to Japanese immigrants who arrived during the rubber boom in the late 19th century in the Mapiri River region; these lineages have since been transmitted within local populations [77]. In Suriname, subgenotype B3 has been linked to Javanese ancestry [78].

Genotype G seems to be driven by more recent transmission dynamics, unlike the lineages discussed above, whose distributions are largely a reflection of historical population movements [79]. The highest number of reported HBV genotype G sequences among LAC countries was observed in Mexico, followed by Colombia, Venezuela, Brazil, and Argentina. This genotype has been linked to contemporary transmission networks—particularly among men who have sex with men (MSM)—where it has been repeatedly identified in transmission clusters and shows elevated prevalence among individuals living with HIV [80,81].

The present study shows a heterogeneous distribution of HBV genotypes of Indigenous American, European, African and Asian origins across LAC, which could present important challenges for regional immunization strategies. Currently, most commercial recombinant HBV vaccines are formulated with HBsAg derived from subgenotype A2 (serotype adw2; GenBank J02205.1) [82], based on the pioneering work of Chilean scientist Pablo Valenzuela and colleagues, who in the early 1980s first achieved the expression and assembly of functional, immunogenic 22-nm HBsAg particles in *Saccharomyces cerevisiae* [83,84]. Here, we found that subgenotype A2 predominated only in Argentina, Cuba, and Panama, whereas non-A2 lineages accounted for 74.3% of the sequences analyzed across LAC, primarily represented by the autochthonous genotypes F and H. These Amerindian clades are among the most phylogenetically divergent lineages described to date, predominantly expressing the adw4 serotype, whereas genotype A expresses adw2 [12,36,38]. This serotypic divergence carries direct immunological relevance, since vaccine-induced protection depends primarily on neutralizing anti-HBs antibodies targeting conformational epitopes within HBsAg [85]. A comparable scenario has already been documented in Indigenous Australians, where standard A2-based vaccination conferred sub-optimal protection against the divergent genotype C4, resulting in breakthrough infections despite antibody titers conventionally considered protective (>10 IU/mL) [86]. To address this challenge, several Asian countries have begun combining universal A2-based immunization with regionally targeted vaccines, particularly genotype C-based formulations [87]. This approach could be of particular interest to explore in LAC, where evaluating whether combining the standard A2 vaccine with formulations incorporating native genotypes F or H truly broadens cross-protective immunity could help optimize long-term protective efficacy and accelerate regional HBV elimination.

Finally, the study has several limitations. The bibliometric analysis was based on publicly available titles and abstracts of articles indexed in PubMed, Scopus, and Web of Science; inclusion of full-text articles may have provided a more granular understanding of specific methodologies. Additionally, as with any text-based approach, certain terms may have multiple meanings, although we sought to reduce this ambiguity by incorporating bigram mapping into our network analysis. Furthermore, our HBV bibliometric search approach was designed specifically around hepatitis B literature; therefore, the lower frequency of metabolic and oncological terms reflects their limited co-reporting within HBV cohorts and should not be misinterpreted as a low prevalence of MASLD or liver cancer, nor as a lack of broader research on these conditions in LAC. In parallel, GenBank records were subject to geographic sampling bias, with over 75% of sequences originating from Argentina and Brazil. Despite these limitations, this study provides a comprehensive assessment of the HBV research landscape in LAC to date. By integrating historical publication trends, identifying areas of opportunity in experimental and metabolic research, and tracing the impact of historical migration on contemporary viral genetic diversity, these findings may help inform future regional research priorities.

## 5. Conclusions

This study provides a comprehensive overview of the evolution, current landscape, and key gaps in HBV research across LAC. Research output remains concentrated in a limited number of countries and is mainly observational, while substantial gaps persist in experimental research and in key clinical areas, including hepatocellular carcinoma, antiviral therapy, and metabolic comorbidities. The high diversity of HBV genotypes and subgenotypes identified in the region—spanning Indigenous American, European, African, and Asian lineages—and the predominance of non-A genotypes underscore the need for region-specific approaches to both vaccination and treatment. Addressing these gaps will likely require greater investment in experimental and translational research, as well as stronger regional collaboration, to support the development of HBV control strategies that are better aligned with the region’s epidemiological and virological context.

## Data Availability

The datasets analyzed in this study were obtained from PubMed, Scopus, and Web of Science, as well as from NCBI Virus (https://www.ncbi.nlm.nih.gov/labs/virus/vssi/; accessed on 25 January 2026). The dataset is publicly available from these sources.

## Supplementary Materials

The following supporting information can be downloaded at https://www.mdpi.com/article/10.3390/pathogens15101025/s1: Supplementary Figure S1: Top 40 First and Last Authors in Hepatitis B Virus Research in Latin America and the Caribbean; Table S1: Genotype and subgenotype assignments of 9528 HBV sequences from Latin America and the Caribbean retrieved from GenBank.
