# Supplementary material for: Mapping Hepatitis B Virus Research and Genomic Diversity in Latin America and the Caribbean
Source: Pathogens. 2026 Sep 30;15(10):1025. doi: 10.3390/pathogens15101025 (PMC13708633; doi:10.3390/pathogens15101025)
Supplement: Supplementary file 1 [file pathogens-15-01025-s001.zip › Supplementary_Figure_S1.pdf]

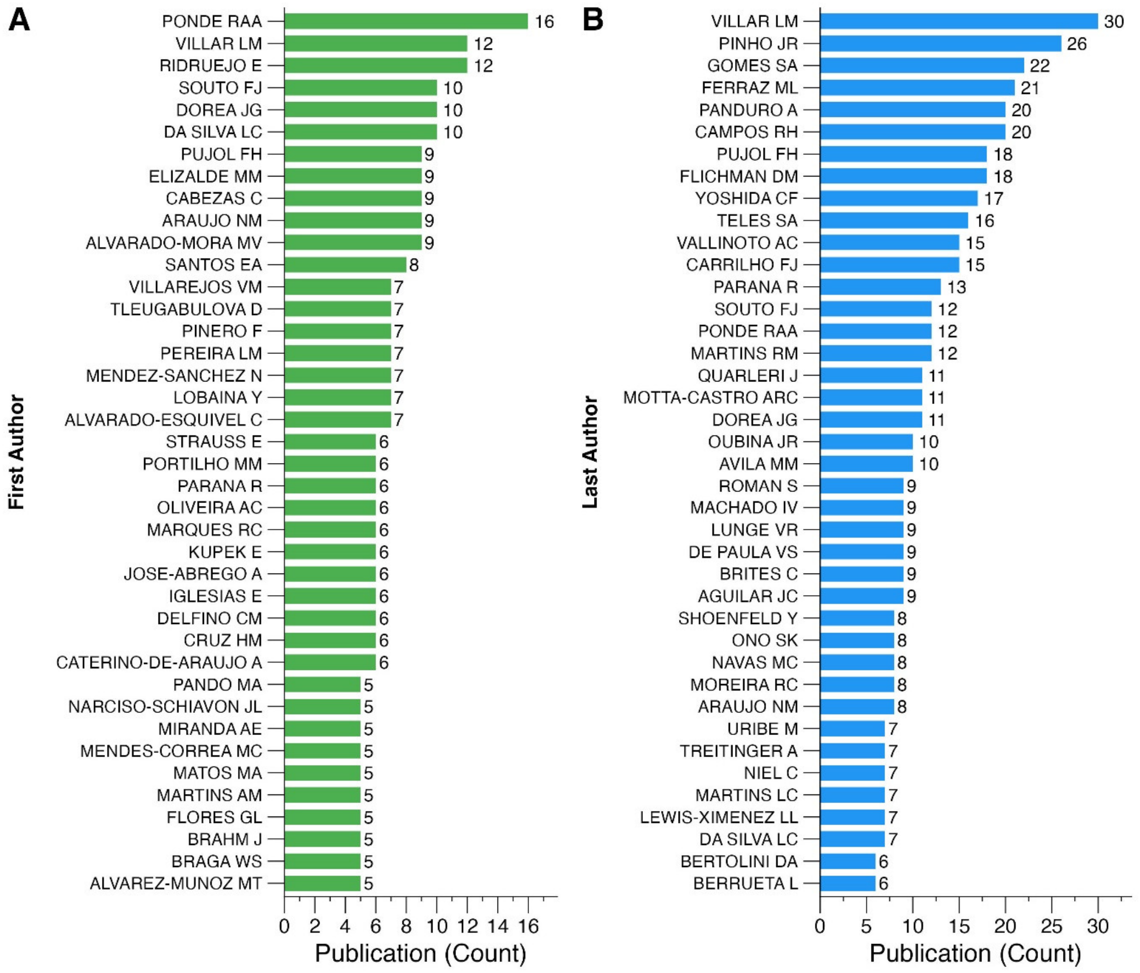

Supplementary Figure S1: Top 40 First and Last Authors in Hepatitis B Virus Research in Latin America and the Caribbean;
